# Purinergic receptor mediated calcium signalling in urothelial cells

**DOI:** 10.1038/s41598-019-52531-9

**Published:** 2019-11-06

**Authors:** Russell Chess-Williams, Donna J. Sellers, Stuart M. Brierley, David Grundy, Luke Grundy

**Affiliations:** 1 Centre for Urology Research, Faculty of Health Sciences and Medicine, Bond University, Gold Coast, Queensland Australia; 2 Visceral Pain Research Group, Centre for Neuroscience, College of Medicine and Public Health, Flinders University, Bedford Park, South Australia 5042 Australia; 3 Hopwood Centre for Neurobiology, Lifelong Health Theme, South Australian Health and Medical Research Institute (SAHMRI), North Terrace, Adelaide, South Australia 5000 Australia; 4 Centre for Nutrition and Gastrointestinal Diseases, Discipline of Medicine, University of Adelaide, North Terrace, Adelaide, South Australia 5000 Australia; 5 Department of Biomedical Science, University of Sheffield, Sheffield, United Kingdom

**Keywords:** Bladder, Calcium signalling

## Abstract

Non-neuronal ATP released from the urothelium in response to bladder stretch is a key modulator of bladder mechanosensation. Whilst non-neuronal ATP acts on the underlying bladder afferent nerves to facilitate sensation, there is also the potential for ATP to act in an autocrine manner, modulating urothelial cell function. The aim of this study was to systematically characterise the functional response of primary mouse urothelial cells (PMUCs) to ATP. PMUCs isolated from male mice (14–16 weeks) were used for live-cell fluorescent calcium imaging and qRT-PCR to determine the expression profile of P2X and P2Y receptors. The majority of PMUCs (74–92%) responded to ATP (1 μM–1 mM), as indicted by an increase in intracellular calcium (iCa^2+^). PMUCs exhibited dose-dependent responses to ATP (10 nM–1 mM) in both calcium containing (2 mM, EC_50_ = 3.49 ± 0.77 μM) or calcium free (0 mM, EC_50_ = 9.5 ± 1.5 μM) buffers. However, maximum iCa^2+^ responses to ATP were significantly attenuated upon repetitive applications in calcium containing but not in calcium free buffer. qRT-PCR revealed expression of P2X_1–6_, and P2Y_1–2_, P2Y_4_, P2Y_6_, P2Y_11–14_, but not P2X_7_ in PMUCs. These findings suggest the major component of ATP induced increases in iCa^2+^ are mediated via the liberation of calcium from intracellular stores, implicating functional P2Y receptors that are ubiquitously expressed on PMUCs.

## Introduction

As the bladder fills, bladder afferents embedded within the detrusor smooth muscle and urothelium provide signals relating the degree of bladder distension into spino-bulbo-spinal reflexes responsible for maintaining continence and supraspinal nuclei for sensory processing^1,2^. Although there are subtypes of bladder afferents that are considered to be tension receptors, thereby directly transducing bladder stretch into neuronal activation^3^, a role for adenosine 5′-triphosphate (ATP) released from the urothelium in response to bladder stretch has also been identified in modulating bladder mechanosensation^4^.

ATP is released from urothelial cells *in-vitro* and *in-vivo* in response to cell or bladder stretch^5–8^, and significant increases in the levels of urothelial ATP release have been detected in pre-clinical models of spinal cord injury, feline interstitial cystitis, and cyclophosphamide induced cystitis^9–12^. Furthermore, enhanced ATP release is also seen from bladder strips isolated from patients with interstitial cystitis/bladder pain syndrome and neurogenic and idiopathic detrusor overactivity^13–15^. The mechanism underlying ATP release from the urothelium has been shown to integrate both traditional vesicular mechanisms^9,16^, as well as direct release via pannexin and connexin channel proteins^17,18^. A number of studies, however, have shown that urothelial ATP release is controlled by a rise in intracellular calcium concentrations, with agents that interfere with intracellular calcium entry or the liberation of inositol triphosphate (IP_3_) able to block stretch induced ATP release^9,10,19–23^. As ATP is released from urothelial cells during stretch and acts on the underlying afferent nerves, there is also the potential for ATP to act in an autocrine manner, modulating urothelial cell function^24–26^.

Two functional subclasses of membrane bound P2 purinergic receptors (P2X and P2Y) mediate the extracellular actions of ATP^27^. Functional P2X and P2Y purinergic receptors have been identified in mouse, rat, and guinea pig urothelial cells, as well as human urothelial cell lines^26,28–30^. P2X receptors (P2X_1_-P2X_7_) are ionotropic ligand gated ion-channels, which with the exception of P2X_7_, are characterised by rapid activation and fast inactivation^31^. P2Y receptors (P2Y_1_, P2Y_2_, P2Y_4_, P2Y_6_, P2Y_11_, P2Y_12_, P2Y_13_, P2Y_14_), in contrast, are classic metabotropic G-protein coupled receptors (GPCRs), coupling with G_q/11_, G_s_ and G_i_ proteins to either activate phospholipase C and release intracellular calcium or bind adenylyl cyclase to modulate cAMP levels^27^. A range of studies, using various techniques and urothelium from cats, rats, and humans have provided evidence that the urothelium expresses a comprehensive repertoire of purinergic receptor subtypes, including P2X_1–7_, and P2Y_1,2,4_^6,28,29^.

The precise role of autocrine purinergic signalling within urothelial cells has yet to be fully determined, however, the maintenance of intracellular calcium homeostasis and further release of neuromodulators is a key consideration. Despite this, only a limited number of studies have systematically explored calcium signalling in urothelial cells. Activation of purinergic receptors upon the urothelium evokes an increase in intracellular calcium which induces acetylcholine release^24^ as well as auto-feedback to influence ATP release itself^13^. Uridine 5′-triphosphate (UTP) has also been shown to significantly enhance ATP release via intracellular calcium pathways^26,28^ indicating that P2Y receptors are an essential component of the urothelial purinergic signalling system.

In this study we provide the first systematic characterisation of extracellular and intracellular calcium contributions to the urothelial response to ATP using primary mouse urothelial cells (PMUCs). Furthermore, we provide the first quantified expression profile of P2X and P2Y receptors in PMUCs and found that intracellular calcium contributes the majority of the functional calcium response to ATP in these cells, implicating P2Y receptors that couple to GPCRs.

## Results

**Figure 1:**
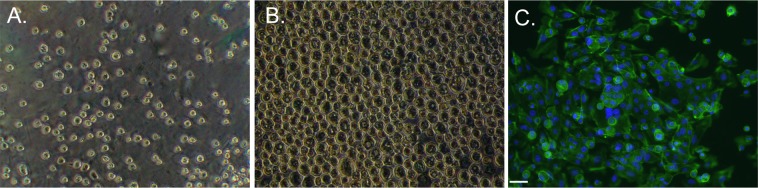
Primary mouse urothelial cells. (**A)** Light microscope images of PMUCs immediately following isolation and plating on collagen IV coated coverslips, and **(B)** after 30 minutes in an incubator (37 °C, 95/5% O_2_/CO_2_). Cells migrate towards each other forming a cell layer. **(C)** Representative confocal image of urothelial cells 24 hrs after isolation incubated with both primary (CK7) and secondary antibodies (AF488) and mounted with Prolong Gold Antifade with nuclei staining positive for 4′6-diamidino-2-phenylindole (DAPI). Cells were excited with 495-nm that emits fluorescence at 505- to 534-nm. Scale bar, 20 μm.

Immediately following plating of the PMUCs onto collagen coated coverslips, the cells were randomly dispersed (Fig. 1A). After 30 minutes, the urothelial cells from the same coverslip had migrated to form a continuous single sheet of cells (Fig. 1B). Primary cultures were confirmed to be of urothelial origin through positive staining with the transitional epithelial cell marker cytokeratin 7 (Fig. 1C).

**Figure 2:**
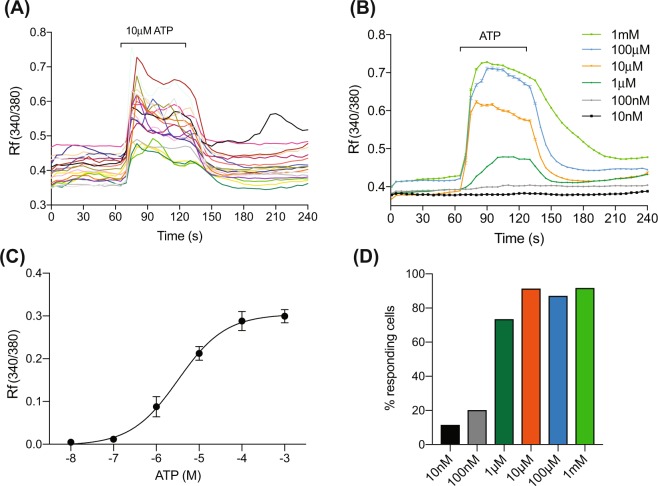
ATP activates urothelial cells. (**A)** Representative trace of a calcium imaging experiment reveals that ATP (10 μM) is able to induce sustained intracellular calcium entry in PMUCs that returns to baseline following washout. Each coloured line represents an individual urothelial cell from a single experiment. **(B)** Group data shows urothelial responses to ATP (10 nM–1 mM) are dose dependent with little or no response to 10–100 nM ATP, but sustained intracellular calcium responses to 1 μM, 10 μM, 100 μM, 1 mM (N = 6, n = 37–76 per concentration, Mean ± SEM). **(C)** Non-linear fit of PMUCs peak response to ATP reveal an EC_50_ value of 3.49 ± 0.77 µM ATP (N = 6). **(D)** Less than 20% of PMUCs respond to 10–100 nM ATP, whereas 74–92% of PMUC’s respond to 1 μM to 1 mM ATP.

Exposure of individual PMUCs to ATP (10 μM) induced a significant rise in intracellular calcium (iCa^2+^) levels, as reflected by an increase in the fluorescent emissions ratio during continuous application (Fig. 2A). PMUCs responded to ATP with variable sensitivity, but the iCa^2+^ response was generally characterised by two distinct phases. There was an initial rapid rise in iCa^2+^ followed by a brief rapid decay, and a more sustained level of iCa^2+^, which was maintained until ATP was removed and a rapid return to baseline calcium levels was observed (Fig. 2A).

PMUC iCa^2+^ responses to ATP were concentration dependent (Fig. 2B,C). Whilst the majority of PMUCs (74–92% of cells/coverslip) respond to high concentrations of ATP (1 μM–1 mM), relatively few (12–20% of cells/coverslip) respond to low concentrations of ATP (10–100 nM) with robust iCa^2+^ transients (Fig. 2D). Responses to ATP are maximal at 100 μM with no further increases upon application of 1 mM ATP (Fig. 2B,C). The EC_50_ for ATP evoked iCa^2+^ responses in urothelial cells was 3.49 ± 0.77 μM.

**Figure 3:**
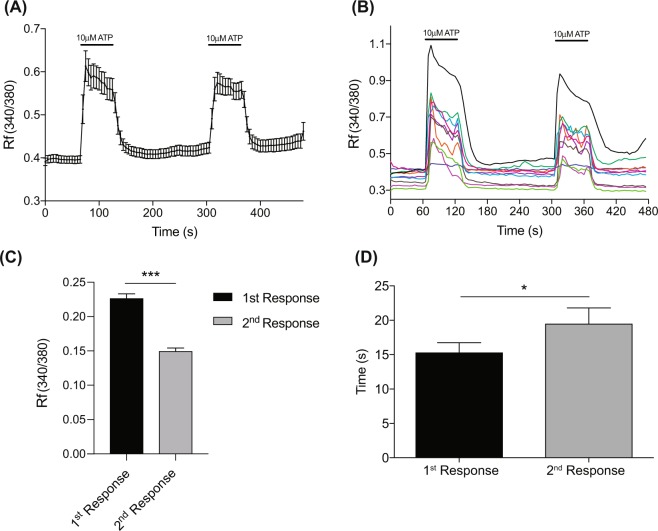
PMUCs respond to repeated applications of ATP. (**A)** ATP (10 μM) evokes a sustained increase in intracellular calcium in PMUCs that rapidly returns to baseline following washout. A second application of ATP also evokes sustained intracellular calcium entry (N = 3, n = 67). **(B)** Representative trace of PMUC calcium fluorescence shows individual responses to repeated application of ATP (10 μM). Each line represents and individual urothelial cell from a single experiment. **(C)** The 2^nd^ response to ATP had a significantly lower peak intracellular calcium entry than the 1^st^ response to ATP (0.22 ± 0.01 vs. 0.15 ± 0.007 Rf340/380, N = 3, n = 67 ***P ≤ 0.001, paired Students t-test). **(D)** The time taken to reach peak intracellular calcium entry is significantly greater during the second response to ATP compared to the first response (15.3 ± 1.4 vs. 19.5 ± 2.4 seconds, N = 3, *P < 0.05, paired Students t-test).

As ATP release from the urothelium is stimulus dependent, such that increases in bladder stretch would evoke a graded increase in ATP concentrations around urothelial cells, we wanted to test the response of PMUCs to repeated applications of ATP. The rise in iCa^2+^ during application of ATP returned to baseline immediately following washout. A subsequent dose of ATP at the same concentration to the same cells also initiated a significant rise in iCa^2+^ (Fig. 3A,B). However, when directly comparing the peak of the 1^st^ and 2^nd^ iCa^2+^ responses to ATP, we observed that the second response, although robust, was significantly attenuated compared to the first response (Fig. 3C). Furthermore, the kinetics of the intracellular calcium response to ATP were altered, with a significant increase in the time taken to reach peak calcium fluorescence with the second ATP application (Fig. 3D).

**Figure 4:**
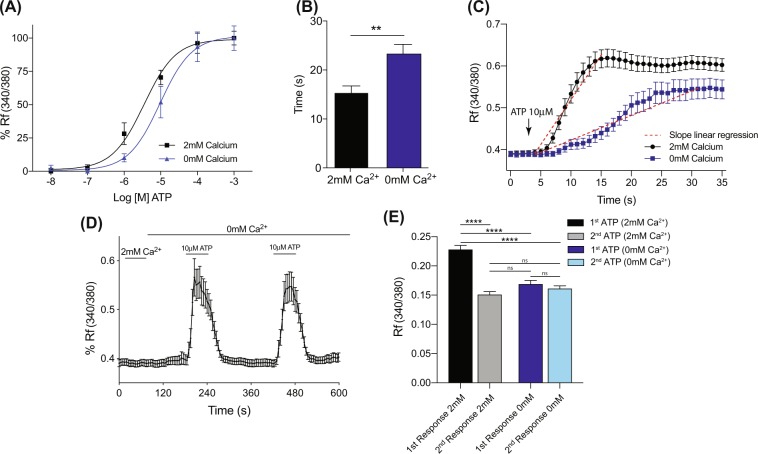
ATP evokes intracellular calcium responses in calcium free buffer. (**A)** Dose response of PMUCs to ATP (10 nM–1 mM) in normal calcium containing extracellular buffer (2 mM) and calcium free buffer (0 mM). PMUCs exhibit a dose-dependent increase in intracellular calcium in nominal calcium buffer, but the EC_50_ to ATP is reduced compared to nominal calcium conditions (3.49 ± 0.77 vs. 9.5 ± 1.5 μM, N = 6, n = 314–360). **(B)** The time taken for ATP (10 μM) to induce peak intracellular calcium in 0 mM calcium buffer is significantly longer than when cells are exposed to ATP (10 μM) in normal 2 mM calcium buffer (15.3 ± 1.4 s Vs 23.3 ± 1.9 s, N = 3, n = 62 **P ≤0 0.01 unpaired t-test). **(C)** The initial kinetics of the intracellular calcium response to ATP (10 μM), calculated by linear regression of the initial slope, is dramatically reduced in the absence of extracellular calcium 0.022 ± 0.0025 Vs 0.0058 ± 0.0007015 dRf/dTime (s) (N = 3, n = 67, n = 62). **(D)** In calcium free buffer, ATP (10 μM) evokes a sustained increase in intracellular calcium in PMUC’s that rapidly returns to baseline following removal of ATP. A second application of ATP in calcium free buffer evokes a sustained intracellular calcium entry (N = 3, n = 62). **(E)** Peak evoked intracellular calcium responses to 10 μM ATP were significantly reduced in calcium free buffer (0.22 ± 0.01 vs. 0.17 ± 0.01 Rf340/380, N = 3, n = 67, n = 62 ***P ≤ 0.001; one-way ANOVA with Tukey’s post-hoc multiple comparisons). Peak evoked intracellular calcium responses were not significantly reduced during duplicate applications of ATP (10 μM) in calcium free buffer (0.17 ± 0.01 vs. 0.16 ± 0.007 Rf340/380, N = 3, n = 62, ns P ≥ 0.05; one-way ANOVA with Tukey’s post-hoc multiple comparisons).

To determine the relative contribution of calcium release from intracellular stores to the PMUC response to ATP, we investigated the response to ATP in a calcium free buffer (Fig. 4). Whilst application of ATP in calcium free buffer evoked a dose dependent increase in iCa^2+^ above baseline (Fig. 4A), there was a rightward shift in the concentration-response curve to ATP in calcium free buffer and a reduction in the EC_50_ from 3.49 ± 0.77 μM in 2 mM Ca^2+^ to 9.5 ± 1.5 μM in 0 mM Ca^2+^ buffer (Fig. 4A). The time taken to reach peak iCa^2+^ fluorescence was also significantly increased in calcium free buffer compared to control solution (Fig. 4B). A closer look at the initial kinetics of the urothelial response to ATP in calcium free buffer reveals a significantly slower increase in iCa^2+^ compared to ATP responses in calcium containing buffer. Because PMUCs took longer to reach peak iCa^2+^ in response to ATP in calcium free buffer, the characteristic two phase iCa^2+^ response seen in control experiments was less obvious, instead replaced by a response which did not exhibit an initial sharp peak (Fig. 4C,D). The maximal intracellular calcium response to ATP in the absence of calcium was significantly reduced compared to control 2 mM calcium buffer (Fig. 4E). Moreover, when comparing duplicate applications of ATP in calcium free buffer, we show that maximum iCa^2+^ responses are not significantly reduced between the 1^st^ and 2^nd^ incubations with ATP (Fig. 4D,E). Additionally, we also observed that the maximal response to ATP in calcium free buffer is similar to the 2^nd^ incubation with ATP in normal calcium buffer (Fig. 4E).

**Figure 5:**
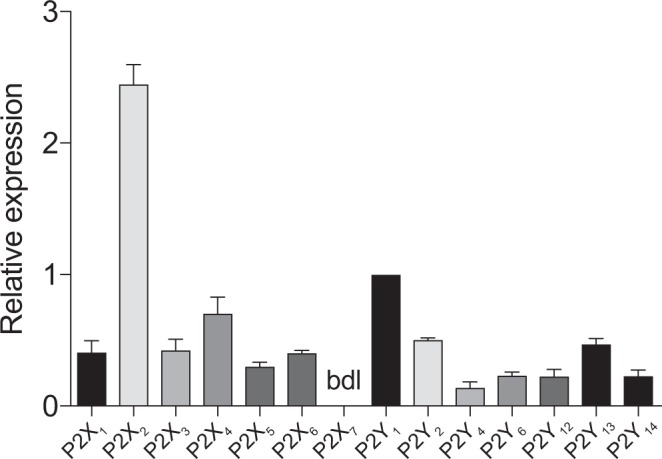
Urothelial cells express P2X and P2Y receptors: PMUCs express P2X and P2Y receptors. mRNA for P2X_1_-P2X_6_, and P2Y_1_, P2Y_2_, P2Y_4_, P2Y_6_, P2Y_11_, P2Y_12_, P2Y_13_, and P2Y_14_ was expressed in PMUC’s. P2X_7_ transcript was below the detection limit (bdl) of the PCR assay. Data presented as expression relative to P2Y_1_ (N = 3).

Membrane bound P2X and P2Y purinoceptors mediate the response to extracellular ATP^27^. Using qRT-PCR we show that PMUCs express almost the complete repertoire of purinergic P2 receptors, with the exception of P2X_7_ which was below the level of detection (Fig. 5). When comparing the expression of P2 receptors relative to the expression of P2Y_1_, we identified that P2X_2_ is the most ubiquitously expressed P2X receptor. The P2Y_1_ receptor was the highest expressed P2Y receptor, followed by P2Y_2_.

## Discussion

Extracellular responses to ATP are mediated by two functional subclasses of membrane bound P2 purinergic receptors, P2X and P2Y. P2X receptors (P2X_1_-P2X_6_) are ionotropic ligand gated ion-channels which show marked desensitisation following rapid activation^31^. P2Y receptors (P2Y_1_, P2Y_2_, P2Y_4_, P2Y_6_, P2Y_11_, P2Y_12_, P2Y_13_, P2Y_14_), in contrast, are GPCRs, mediating effects via intracellular signalling pathways^27^. Data from the current study provide a number of novel findings that have implications for understanding the autocrine signaling of the bladder urothelium in response to ATP.

In this study we provide the first quantitative expression profile of P2X and P2Y receptors of the urothelium. We found the most significant expression of P2X_2_, P2X_4_, P2Y_1_ and P2Y_2_, as well as lesser expression of other purinergic receptor subtypes including P2Y_6_. There are many benefits in the use of PMUC’s versus a known urothelial cell line, however, a small possibility exists that our PMUC culture is not 100% pure. Nonetheless, our dissection and culture has been refined to ensure the highest purity, and this technique has been used extensively for the purpose of characterising functional urothelial responses^5,8,30^. In addition, our immunostaining for CK7 indicates that our PMUC culture is pure and our data is largely consistent with previous reports of urothelial purinergic receptor expression^28,29,32,33^. P2X_7_ mRNA was not detected in our study, consistent with a lack of expression in human urothelium^29^. P2X_7_ is predominantly expressed on cells of hematopoietic lineage as well as glial cells, Schwann cells and astrocytes^34^. An immunohistochemistry study of cat urothelium, has previously revealed significant P2X_7_ staining throughout the basal and apical layers of the urothelium^6^, however, more recent studies have identified the urothelium is prone to non-specific adsorption of antibodies^35,36^. To ultimately determine the precise molecular architecture of the purinergic receptors expressed on the urothelium, a comprehensive analysis using multiple complementary techniques, including qPCR, western blot, immunohistochemistry and *in-situ* hybridization will be required.

Using live cell calcium imaging, we have systematically characterised the functional response of primary mouse urothelial cells to ATP, revealing a key role for intracellular calcium stores in urothelial ATP responses. Consistent with our observations, a number of previous studies have shown functional responses to ATP in isolated urothelial cells from mouse, rat, and guinea pig^26,28–30^. In the current study, the urothelial response to ATP was characterised by a rapid rise in intracellular calcium, followed by sustained intracellular levels of calcium in the presence of the agonist. Following duplicate applications of sub-maximal ATP, and in a calcium free extracellular solution, the magnitude and kinetics of ATP evoked responses were altered. As the repeat sub-maximal doses of ATP were applied with only a short washout period, it is possible that the changes in the observed response are due to alterations in the function of urothelial purinergic receptors responsible for calcium influx. As P2X receptors undergo rapid desensitisation, it is likely that the reduction in response that we observed during duplicate application of ATP in calcium containing buffer is due to a desensitisation of these P2X receptors. P2X receptors also exhibit rapid activation kinetics, with direct influx of cations across the electrochemical gradient responsible for intracellular calcium influx. We, like others, found significant expression of the P2X_2_ receptor in the urothelium^6,37,38^, as well as expression of P2X_1,3,4,5,6_ which have also previously been identified within the urothelium^6,37–39^. Therefore, if these receptors are desensitised we would expect, and in this study observed, an increase in the time taken to reach peak intracellular calcium during a second application of ATP. Furthermore, the obvious differences in the rate of intracellular calcium rise that occurred in calcium free, compared to calcium containing buffer further implicate P2X receptors in the initial fast component of intracellular calcium influx in response to ATP. The relative abundance of P2X_2_ over other P2X receptors suggests this receptor may be a key integrator of this response, however, in a somewhat related function, P2X_4_ mediates ATP-induced calcium influx in response to fluid shear stress in human vascular endothelium^40^ and its role in urothelial evoked calcium influx cannot be currently ruled out. The P2X receptor isoforms mediating this initial ATP response requires further elucidation. Together these data support a mechanism whereby liberation of intracellular calcium via a slower G-protein coupled mechanism involving inositol triphosphate (IP_3_)^41^, rather than direct influx across the electrochemical gradient, may be responsible for the intracellular calcium influx in response to repeated applications of ATP^27^. P2Y_1_, P2Y_2_, P2Y_4_, P2Y_6_, P2Y_11_, couple to phospholipase C and the liberation of intracellular calcium via IP_3_ and we, like others, identified the expression of these P2Y receptor subtypes within the urothelium^6,26,28^.

In further support of a P2Y mediated ATP response in the urothelium, we identified that ATP evoked increases in intracellular calcium persist even when cells are superfused in a calcium free extracellular solution. Removal of extracellular calcium from the perfusion buffer isolates the GPCR mediated P2Y receptor response from the ionotropic P2X component of calcium influx^42^, and thus provides additional evidence that a large proportion of the urothelial response to ATP is mediated by the liberation of calcium from intracellular stores rather than through membrane bound calcium channels. In addition, with repeated applications of ATP in the absence of extracellular calcium, there was no reduction in peak response or obvious change in response profile. In rat cultured urothelial cells, UTP, an agonist of P2Y receptors, has been shown to stimulate intracellular calcium rises via a phospholipase C-linked mechanism which was unaffected by extracellular calcium but significantly attenuated by store depletion^26,28^. Our study also provide some insight into the mechanisms responsible for urothelial calcium conductance. In normal calcium containing buffer, our data suggests extracellular calcium conductance provides the initial fast component of the intracellular calcium response to ATP, likely via non-selective P2X receptor cation channels, corroborating computational modelling and functional studies of urothelial cell calcium signalling that implicated extracellular channel currents^43,44^. During duplicate applications of ATP in calcium free buffer we observe calcium responses that are of equivalent magnitude in the first and second application, as well as a rapid return to baseline following ATP removal. These data suggest that after removal of ATP as a stimulus, intracellular calcium is not lost in significant amounts into the extracellular space and that intracellular calcium is rapidly and efficiently sequestered back into the endoplasmic reticulum for future use.

The ability of urothelial cells to respond to continuous or repeated stimuli with an increase in intracellular calcium, the known stimulus for distension evoked ATP release from the urothelium^22,23^, is essential to the proposed physiological role of urothelial signalling during bladder distension. The importance of ATP in providing autocrine modulation of intracellular calcium levels within the urothelium is implicated by the role of intracellular calcium, via the liberation of IP_3_, in mediating stretch evoked ATP release^19,22,23^, the ability of ATP to induce ATP release^13^, and the multitude of interactions that ATP has been proposed to mediate in the sub-urothelium relating to mechanosensitivity^17,27,45–47^. Thus, a mechanism by which the actions of ATP and its metabolites are able to mediate further ATP release could be an additional mechanism contributing to the enhancement in reflex bladder activity observed in a number of bladder disorders. Indeed, ATP release is enhanced from bladder strips isolated from neurogenic and idiopathic detrusor overactivity patients, as well as IC/BPS patients^13–15^, and an increased urinary content of ATP is observed in women with OAB^48^. Whilst this ATP is likely to be acting on underlying bladder afferent nerves^49,50^, bladder sensations could be further modified by autocrine actions of ATP on urothelial cells.

The presence of ectonucleotides in the urothelial layer^51^, which have the ability to breakdown ATP to adenosine-5′-diphosphate, a potent agonist of P2Y_1_, and results showing that both ADP and UTP are able to stimulate release of ATP^52^, have all provided further credibility to the theory that P2Y receptors have an essential role in urothelial function and ATP release. Intriguingly, the ATP metabolite adenosine, acting through P1 receptors has been shown to inhibit further ATP release and this was proposed to be through inhibition of intracellular calcium liberation^19^. A systematic assessment of the contribution of purinergic receptors in the response to ATP will be an important area for future investigation.

These results have shown for the first time that the major component of ATP induced increases in urothelial intracellular calcium are via the liberation of calcium from intracellular stores, implicating but not confirming functional P2Y receptors. In addition, these results provide the first complete expression profile of P2X and P2Y receptors on PMUCs. The control of urothelial intracellular calcium levels is a necessary factor in ATP release, and ATP release is an essential component in the control of micturition within the bladder.

## Methods

The methods described have been used in previous studies and were performed as previously described^5,8,53^. Comprehensive details of the methods are provided to account for any minor variations in protocol.

### Animals

The University of Sheffield Animal Care Committee (UK) approved experiments involving animals under a project license issued in accordance with the UK Animals (Scientific Procedures) Act 1986. Adult (14–16 weeks) C57BL/6J male mice were used in this study. Mice were group housed (5 mice/cage) in specific housing rooms within a temperature-controlled environment of 22 °C and a 12:12 hr light-dark cycle. Mice had free access to food and water at all time. All experiments were performed on cells isolated from mice that were humanely euthanized by cervical dislocation in accordance with the guidelines set-out by the UK Animals Act 1986^5,8^.

### Isolation of primary mouse urothelial cells (PMUCs)

Culture of primary mouse urothelial cells was performed as previously described^5,8,30^. Following cervical dislocation, bladders were excised from the mouse, dissected in sterile PBS and pinned urothelial side up in a SYLGARD^™^ coated dish. The bladder was incubated with 2.5 mg/ml Dispase dissolved in modified Eagle’s medium (MEM) media (Gibco) containing 1% antibiotic-antimycotic (PSF) solution (Gibco) and 0.7% Hepes (1 M) for 3 hrs at room temperature (21 °C). Cells were gently scraped from the urothelium using a blunt scalpel and dissociated in 0.025% trypsin-EDTA (Invitrogen) at 37 °C for 10 mins using gentle trituration with a Pasteur pipette at 5 and 10 minutes. The cell suspension was resuspended in MEM with 10% Fetal Bovine Serum (FBS) before centrifugation (15 min, 1,500 rpm, 4 °C). The MEM + FBS was aspirated and the cell pellet was resuspended in fresh keratinocyte serum free media (KSFM; Invitrogen) before being plated on collagen (IV) (Sigma-Aldrich) coated coverslips. Coverslips were left for 4 h in an incubator at 37 °C and 5% CO_2_ before flooding with KSFM (2 ml/well).

### Calcium imaging of cultured urothelial cells

Calcium imaging of PMUC’s was performed as previously described^5,8^. Cultured urothelial cells (20–24 hrs) attached to coverslips were loaded with 2 μM Fura-2-acetoxymethyl ester (Fura-2AM; Sigma Aldrich) for 15 minutes in the dark at 37 °C. Coverslips were then placed in a washing well containing KSFM media at 37 °C for 15 minutes before being washed in HEPES buffer (Composition in mM (NaCl 142, NaHCO3 5, HEPES 10, Glucose 16, KCL 2, CaCl2 2, MgCl2 1, 0.1% BSA, 310 mOsm) at room temperature for 15 minutes before imaging. Coverslips containing cells were transferred to a perfusion chamber mounted on an inverted microscope (Axiovert S100 TV, Zeiss, Cambridge, UK) equipped with a 20x oil immersion objective (Zeiss). Cells were continually superfused with external HEPES solution at a rate of approximately 1.5 mL/min. Cells were alternately illuminated at 340 and 380 nm with a 20 msec exposure time (Polychrome IV, TILL Photonics, Munich, Germany). Emitted light was passed through a 510 nm band pass filter and collected by a 512B Cascade CCD camera (Photometrics, Tucson, AZ) and images were acquired at 0.5 Hz. MetaMorph imaging software (Molecular Devices, Sunnyvale, CA) was used to analyse all calcium imaging experiments.

### Calcium imaging protocol

Cells for experiments carried out in normal calcium (2 mM) containing buffer were exposed to an individual concentration of ATP (10 nM–1 mM) for 60 seconds via continual perfusion. If a second dose of ATP was to be applied, a 3-minute washout with HEPES was allowed, followed by ATP at the same concentration for a further 60 seconds. Individual cells were easily discriminated based on fluorescent intensity under the microscope. For experiments in calcium free (0 mM) HEPES (Composition in mM (NaCl 142, NaHCO3 5, HEPES 10, Glucose 16, KCL 2, MgCl_2_ 3, 0.1% BSA, 310 mOsm), recordings were started in calcium containing HEPES and switched to continual perfusion with calcium free HEPES during the recording period for two minutes prior to addition of ATP to ensure a complete switch in solution.

### Immunohistochemistry of cultured urothelial cells

Immunohistochemistry and microscopy of cultured urothelial cells was performed as previously described^5^. Urothelial cells were labeled for transitional epithelium using monoclonal antibody cytokeratin 7 (CK7) (OV-TL 12/30; ThermoFisher). The details of the primary antibody used are in Table 1. Coverslips were washed with 0.1 M phosphate-buffered saline (PBS) three times and fixed with ice-cold 4% PFA at 4 °C for 45 minutes. Coverslips were washed with saponin 0.05% (Sigma-Aldrich) + 2% FBS in 0.1 M PBS (SF-PBS) to remove excess PFA and permeabilise cell membranes. Nonspecific binding of secondary antibodies was blocked with 3% bovine serum albumin diluted in 0.05% SF-PBS (Sigma-Aldrich) for 1 h. Coverslips were incubated with primary antisera and diluted in SF-PBS overnight (28 h) at 4 °C. Sections were then washed 3x in PBS and incubated in the dark for 2 h at room temperature with secondary antibody conjugated to Alexa Fluor. Cells were then washed in SF-PBS before mounting in Prolong Gold Antifade with DAPI (ThermoFisher Scientific) and coverslipped. Slides were allowed to dry for 24 h before visualization.

**Table 1:** Primary and secondary antisera details.

	Species Raised in	RRID/AF Conjugate	Manufacturer	Dilution
*Primary antigen Cytokeratin 7 (clone OV-TL 12/30)*	Mouse	AB_10989596	ThermoFisher	1:50
*Secondary Antigen Mouse IgG1*	Goat	488	ThermoFisher	1:1000

### Microscopy

Fluorescence was visualized with a confocal laser scanning microscope (Leica TCS SP8X; Leica Microsystems, Wetzlar, Germany). Images (1,024 × 1,024 pixels) were obtained using a X63 lens (software zoom X1.3) X60 oil immersion objective, and sequential scanning (4- to 5-line average). Separation of fluorophores was achieved using white line laser tuned to 495-nm excitation and 505- to 534-nm emission detection settings for AF-488 and 405-nm excitation and 425- to 475-nm emission detection settings for DAPI. Confocal settings were optimized to reduce background staining by adjusting the white light laser intensity, emission window (as described above), and amplifier gain [726.7 offset: X0.07 (AF488); 10 offset: X0.1 (DAPI)]. These settings were saved and used for all imaging^5^.

### qRT-PCR of urothelial cells

Following isolation of PMUC, cells were incubated at 37 °C in KSFM media for 24 hrs. mRNA from PMUC’s was isolated (RNAeasy minikit, Qiagen) and cDNA was synthesized by reverse transcription using superscript III (Invitrogen) from mRNA following the manufacturers protocol. cDNA was amplified by PCR for 35 cycles (Research Rotor-Gene 6000 real time thermocycler; Corbett-Qiagen) with forward and reverse primers (Table 2) and iQSYBR Green Master Mix (Biorad). Primers were designed as exon spanning with a product size of between 98–207 bp and Tm°C of less than 65 °C (Table 2). All PCR reactions were made up to a total of 25 μl, cyclic conditions were set at: 95 °C for 12 minutes as an initial hold stage followed by 40 cycles of 95 °C for 30 s, 59 °C for 30 s, 72 °C for 30 s, followed by a melt curve of 0.5 °C increments every 30 seconds from 72–95 °C. All samples were assayed in triplicate in the same plate. The relative amount of a target gene was calculated by the 2^−ΔΔCt^ method using β-actin as a housekeeping gene.

**Table 2:** Primer sequence, product size, and Tm for purinergic receptors investigated in this study.

Gene		Primer Sequence 5′- 3′	Product	Position	Tm°C
P2X1	Forward	CAAGTATGCGGAGGACATGG	131 bp	1362–1493	58.4
	Reverse	CACACTGAGTCAAGTCCGG			57.8
P2X2	Forward	CCATCAGGTGAAGGACCAGC	118 bp	1454–1571	60.4
	Reverse	GCTGGTCAAGAGTGTCCACC			60.6
P2X3	Forward	CAGTGTTCACCAGTGACCAGGC	128 bp	1086–1213	63.6
	Reverse	GCCTGTTGAAGGTTCTGCAGCC			64.4
P2X4	Forward	CGTCTGTCACTCTAGAGACGG	202 bp	1229–1431	59.3
	Reverse	GGTGCTGTTATGGACGTGTGG			61.5
P2X5	Forward	GTAGCCAGAGCTCTTGGCAGG	115 bp	1494–1608	62.8
	Reverse	CTCAGAAGCCACATCCTGAGC			60.8
P2X6	Forward	GACCTGCTGCTACTGTATGTGG	104 bp	1120–1226	60.7
	Reverse	GGCTCGGTCTATGAACTGTTGG			61
P2X7	Forward	CACATTCGCATGGTGGACCAGC	98 bp	1213–1310	64.5
	Reverse	GACAGGTCGGAGAAGTCCATCTGG			64.3
P2Y1	Forward	GTCTCAACAGCTGTGTGGACC	206 bp	1598–1803	61
	Reverse	CTCAGGAGCTAGGATCTCGTGC			62
P2Y2	Forward	CACGATGGACTTAGCTCAGAGG	207 bp	1864–2070	60.2
	Reverse	CAGGAGGCAGAGATAACAGGC			60.2
P2Y4	Forward	CAGCAGCTATGCAGAGGTAGC	194 bp	553–746	60.9
	Reverse	CCTCTGCCTGCAGTTAGTCC			60.1
P2Y6	Forward	GGCAACTGGTCAATTCATGC	150 bp	1583–1732	58
	Reverse	CACATCCTGAGATGTCTAGC			55
P2Y12	Forward	GTGTCAACACCACCTCAGCC	149 bp	420–568	61.1
	Reverse	CCTCATTGCCAAGCTGTTCG			59.8
P2Y13	Forward	GTTCCTCAAGATCATCATGCCG	210 bp	436–645	59.4
	Reverse	GTGTGACTGACCACCTGATGC			61.2
P2Y14	Forward	GTCACGAAGATACAGTGCATGG	131 bp	792–922	59.4
	Reverse	GTGATGGCCGTGTAGAAGACG			61

### Data analysis and statistics

PMUCs intracellular calcium flux was calculated as a ratio between the fluorescent signal at 340/380 nm (e.g. Rf 340/380) for responding cells. All data are presented as Mean ± SEM. Initial slope for urothelial activation kinetics was calculated using a linear regression of time from ATP application to max peak (Rf340/380). For qRT-PCR expression, levels of each target gene were calculated relative to the housekeeping gene, β-actin, and represented relative of P2Y_1_ receptor expression. Statistical analysis was carried out using either paired or un-paired Student’s t-test as appropriate. Statistical significance was confirmed at P < 0.05 using GraphPad Prism 7 software. (N = number of mice, n = number of cells)^53^.

## Data Availability

The datasets generated during and/or analysed during the study are available from the corresponding author on reasonable request.
